# Reconceptualizing Symbolic Magnitude Estimation Training Using Non-declarative Learning Techniques

**DOI:** 10.3389/fpsyg.2021.638004

**Published:** 2021-04-06

**Authors:** Erin N. Graham, Christopher A. Was

**Affiliations:** Department of Psychological Sciences, Kent State University, Kent, OH, United States

**Keywords:** math education, number line, implicit memory, declarative memory, vanishing cues, errorless learning

## Abstract

It is well-documented that mathematics achievement is an important predictor of many positive life outcomes like college graduation, career opportunities, salary, and even citizenship. As such, it is important for researchers and educators to help students succeed in mathematics. Although there are undoubtedly many factors that contribute to students' success in mathematics, much of the research and intervention development has focused on variations in instructional techniques. Indeed, even a cursory glance at many educational journals and granting agencies reveals that there is a large amount of time, energy, and resources being spent on determining the best way to convey information through direct, declarative instruction. The proposed project is motivated by recent calls to expand the focus of research in mathematics education beyond direct, declarative instruction. The overarching goal of the presented experiment is to evaluate the efficacy of a novel mathematics intervention designed using principles taken from the literature on non-declarative learning. The intervention combines errorless learning and structured cue fading to help second grade students improve their understanding of symbolic magnitude. Results indicate that students who learned about symbolic magnitude using the novel intervention did better than students who were provided with extensive declarative support. These findings offer preliminary evidence in favor of using learning combination of errorless learning and cue fading techniques in the mathematics classroom.

## Introduction

It is well-documented that mathematics achievement is an important predictor of many positive life outcomes like college graduation (Adelman, 2006; National Mathematics Advisory Panel, 2008), career opportunities (Moses and Cobb, 2001; Howell and Walkington, 2019), salary (Rose and Betts, 2004), and even citizenship (Education Commission of the States and United States., Dept. of Education, 1998). As such, it is important for researchers and educators to help students succeed in mathematics. Unfortunately, the results from the 2017 National Assessment of Educational Progress (the largest continuing assessment of United States student achievement) suggests that only 40% of 4th grade students met or exceeded proficiency level in mathematics achievement. Even more startling, 20% percent of 4th grade students failed to meet the standards for even basic level of mathematics understanding (National Center for Education Statistics, 2017). Statistics such as these have driven the national agenda to discover and empirically assess the effectiveness of techniques to improve students' mathematics achievement and have fueled increasing research into mathematics interventions. Although there are undoubtedly many factors that contribute to these statistics, much of the research and intervention development has focused on instructional techniques. Indeed, even a cursory glance at many educational journals and granting agencies reveals that there is a large amount of time, energy, and resources being spent on determining the best way to convey information through direct instruction or declarative support. The proposed project is motivated by recent calls to expand the focus of research in mathematics education beyond these standard techniques (Vinter et al., 2010; Indahl, 2015). The overarching goal of the presented study is to evaluate the efficacy of a novel mathematics intervention which combines the principles of errorless learning and structured cue fading to help second grade students improve their understanding of symbolic magnitude.

Although an exhaustive overview of the literature regarding the theory and measurement of symbolic magnitude representations is beyond the scope of this article (see Siegler, 2016 or Kim and Opfer, 2017 for review), it is important to provide some background information in order to better contextualize this study. To start, symbolic magnitude representation refers to one's ability to create and utilize a cognitive structure that maps symbolic numbers (typically Arabic numerals) onto the abstract spatial-numerical quantities that the symbolic number is supposed to represent. Although a broad variety of cognitive structures are possible, most researchers describe the ideal cognitive structure as one that is both evenly-spaced and generally linear in nature; this is called the “mental number-line” (Siegler, 2016). A well-calibrated mental number-line is thought to help individuals more accurately reason about the relationships between numbers and magnitude more broadly (Siegler, 2016; Kim and Opfer, 2017). Unfortunately, the vast majority of both children and adults have deficiencies in their representations of symbolic numbers, and this can lead to errors in numerical reasoning and mathematics performance. Instead of a purely linear representation, young children (and even adults in unfamiliar contexts) tend to spatially overestimate the magnitude of smaller or more familiar quantities and condense larger or more unfamiliar quantities; this is often modeled as a logarithmic representation (Siegler and Opfer, 2003; Friso-van den Bos et al., 2015; Hamdan and Gunderson, 2017). It is hypothesized that learners shift from a logarithmic representation to a more linear representation as they mature and gain familiarity with symbolic numbers, and that this shift from logarithmic to linear representational structure results in a better understanding of the relationships between numbers and symbolic magnitude more broadly (Booth and Siegler, 2008; Holloway and Ansari, 2009; Friso-van den Bos et al., 2015; Hamdan and Gunderson, 2017).

The most common way for researchers to measure representations of symbolic magnitude is through number-line estimation tasks (Siegler and Opfer, 2003). In the most common instantiation of these tasks, learners are presented with a blank, bounded number-line (e.g., a number-line where only the zero point and the end point are marked and given symbolic labels) and asked to mark the location of a given symbolic number. The degree to which individuals are able to accurately complete this task is considered a metric of the quality of their symbolic magnitude representations or the accuracy of their mental number-lines. In addition to their academic utility in helping researchers study how people cognitively represent numbers, number-line estimation tasks also have a more practical utility. First, there is a large literature suggesting that accurate performance on number-line estimation tasks is highly predictive of later achievement in mathematics (Booth and Siegler, 2008; Fazio et al., 2014; Watts et al., 2014; DeWolf et al., 2015; Friso-van den Bos et al., 2015; Schneider et al., 2017). This has led researchers like Booth and Siegler (2006) to assert that the development of a linear mental number-line is critical for student success (however, see Cohen and Sarnecka, 2014 or Muldoon et al., 2013 for disagreement regarding this point). Second, a growing body of research also suggests that number-line estimation (and by extension, the quality of symbolic magnitude representations) is a malleable skill that is responsive to interventions (Ramani and Siegler, 2008; Whyte and Bull, 2008; Opfer et al., 2016; Wall et al., 2016). In perhaps the most famous demonstration of this fact, Ramani and Siegler (2008) used a specially developed, spatial-numerical board game to help improve the number-line estimation skills of students enrolled in a Head-Start program. Results indicated that children who spent time playing the board game, particularly with adult feedback, were better at a subsequent number-line estimation task and had higher performance in other key numerical competencies like numeral identification, magnitude comparison, and counting. The benefits of the intervention were also durable, with students seeing benefits from the interventions over 9 weeks later in number-line estimation, numeral identification, magnitude comparison, and counting. Taken together, these findings suggest that accurate symbolic magnitude representations are both critical to students' math achievement and responsive to interventions; this makes the domain ideal for further study and intervention.

It should be noted that many of the interventions developed to help students improve their symbolic magnitude estimation skills are reliant on declarative memory processes and direct instruction. For example, additional research on the board game intervention developed by Ramani and Siegler (2008) suggests that many of the benefits arise from directly instructing students to engage in a declarative counting on strategy, where students verbalize relevant symbolic numbers in order as they progress through the game. While interventions based on declarative learning techniques are doubtlessly beneficial, they are not without limitations. From a practical standpoint, interventions which require instructor guidance in order to produce benefits are not well-suited for large classrooms where such individualized attention may not always be feasible. From a theoretical standpoint, interventions which rely on declarative memory place large demands on students' working memory resources (Anderson et al., 1996; Ashcraft and Krause, 2007; Oberauer, 2009), which can be especially problematic for domains like mathematics which already place a heavy burden on students' working memory resources. Indeed, there is growing evidence that acquiring and using symbolic magnitude representations recruits working memory resources, especially for young children (Wang et al., 2015).

This combination of working memory demands from both the learning domain and the structure of the intervention likely diminishes the efficacy of the intervention for many at-risk students. For example, research suggests that children and adolescents from lower-SES households underperform their peers on working memory assessments (Herrmann and Guadagno, 1997; Farah et al., 2006; Noble et al., 2007; Evans and Schamberg, 2009; Sarsour et al., 2011; Hackman et al., 2015; Leonard et al., 2015) and that girls suffering from stereotype threat experience a situational decrement in working memory function in math tasks (Bonnot and Croizet, 2007). Moreover, many individuals with learning disabilities have comorbid deficits in working memory processing (Siegel and Linder, 1984; Siegel and Ryan, 1989; Hitch and McAuley, 1991; Swanson, 1993; McLean and Hitch, 1999; Keeler and Swanson, 2001; Wilson and Swanson, 2001; Geary et al., 2007; Andersson, 2008; Swanson et al., 2008; Jitendra et al., 2016) and, in line with more general research on anxiety and working memory (e.g., Eysenck, 1992; Eysenck and Calvo, 1992), many researchers characterize math anxiety as a situational decrement in working memory processing (Ashcraft and Kirk, 2001; Ashcraft and Ridley, 2005; Ashcraft and Krause, 2007). As such, these students might be better served by employing interventions which place fewer demands on working memory resources. We hypothesize that one way to reduce the working memory demands of a mathematics intervention is to reduce its reliance on declarative memory processes. For the purposes of this study, we will attempt to reduce reliance on declarative memory processes through the use of two non-declarative learning techniques: errorless learning and cue fading.

Before moving on to the specific non-declarative learning techniques employed by this study, a point of clarification is required. In much of the cognitive psychology literature, the terms procedural learning and procedural knowledge are used to describe the acquisition and representation of cognitive operations which can be used to facilitate skilled behavior in the absence of declarative activation. However, the terms procedural knowledge and procedural learning mean something different in the mathematics education literature. Procedural knowledge in mathematics education typically refers to the knowledge of the skills, sequences, or steps necessary to solve a mathematics problem (Hiebert, 1986; Rittle-Johnson and Siegler, 1998; Canobi, 2009; although see Star, 2005, 2007). In contrast, conceptual knowledge is described as abstract knowledge about underlying principles and relational structures (Hiebert, 1986; Canobi, 2009; Prather and Alibali, 2009; Crooks and Alibali, 2014). There is considerable debate about how these two constructs develop, with some arguing that procedures must be acquired first (Baroody and Gannon, 1984; Baroody and Ginsburg, 1986; Sun et al., 2001), some arguing that concepts must be acquired first (Donlan et al., 2007), and others arguing that the two constructs influence each other and develop in an iterative fashion (Gelman and Gallistel, 1978; Rittle-Johnson and Siegler, 1998; Rittle-Johnson and Alibali, 1999; Rittle-Johnson et al., 2001, 2015; Rittle-Johnson and Koedinger, 2009; Rittle-Johnson, 2017). Most relevant for our purposes, the conceptual vs. procedural debate in mathematics does not map cleanly onto the declarative vs. procedural distinction in cognition (Kalra, 2015). For example, conceptual knowledge includes both information which can be easily verbalized or accessed explicitly and information which cannot be easily verbalized or accessed implicitly (Rittle-Johnson et al., 2001; Rittle-Johnson, 2017); in contrast, declarative knowledge can only be accessed explicitly and is often easily verbalized (Anderson, 1993). Moreover, procedural knowledge in mathematics can be represented verbally and is often evaluated using explicit recall of problem solving steps (Rittle-Johnson et al., 2001; Kalra, 2015; Rittle-Johnson, 2017); procedural knowledge in the ACT-R framework is represented as automatic, if-then cognitive operations which place few demands on working memory resources (Anderson, 1993). Thus, it is possible to both have conceptual knowledge which is represented in procedural memory and have procedural knowledge which is represented in declarative knowledge. This confusion over terminology is the primary reason for our use of the terms “declarative learning” and “non-declarative learning” in this paper.

Perhaps one of the more robust findings in the non-declarative learning literature is the benefit of errorless learning (Baddeley and Wilson, 1994; Hunkin et al., 1998; Tailby and Haslam, 2003; Anderson and Craik, 2006; Page et al., 2006; Warmington et al., 2013; however see Evans et al., 2000). Errorless learning, as the name suggests, is a technique which minimizes or eliminates the number of errors experienced by the learner during initial learning in hopes that it will improve subsequent knowledge quality and duration. Specifically, it is argued that correcting errors during learning requires learners to deliberately access episodic representations of prior experiences with relevant material, update their knowledge representations to include correct information, and inhibit the re-activation of erroneous material or irrelevant information; each of these processes require substantial working memory involvement (Baddeley, 1992; Baddeley and Wilson, 1994). As such, the reduction of participant errors (especially early on in the learning process) can reduce working memory demands incurred during learning (Baddeley and Wilson, 1994). The present intervention will seek to reduce errors during initial learning in an effort to reduce working memory demands.

Cue fading represents a broad class of learning techniques which seeks to obtain the learning benefits associated with generation (namely long-term retention and transfer to novel context) while reducing the potential for misconceptions or errorful intrusions. There are many ways to instantiate cue fading (Glisky, 1992, Wolery et al., 1992; Riley and Heaton, 2000; Gardner et al., 2011; Fyfe et al., 2014; Hesser and Gregory, 2015; Suh et al., 2020), but the general procedure involves the presentation of some-sort of partial cue or memoranda to which the participant must produce the remainder of the cue or the correct associated response. Although cue fading is not an exclusively non-declarative technique (see Fyfe, McNeil, Son, and Goldstone for a discussion of concreteness fading with explicit declarative support) it can be used to direct a learner's attention to important stimulus features without relying on declarative instruction. One example of non-declarative cue fading is the vanishing cue technique. In this technique, participants begin the task with all possible cues which are then gradually faded (either through physical cue removal or functionally through a decrease in cue utility) until the participant can generate the desired response without support (Glisky, 1992; Riley and Heaton, 2000; Atkinson et al., 2003). Empirical support for the vanishing cue technique is mixed, with some researchers finding evidence for successful interventions in standard word string learning tasks (Tailby and Haslam, 2003), computer vocabulary learning (Glisky et al., 1986; Glisky, 1992), and fading worked examples (Moreno et al., 2006; Salden et al., 2009). However, a meta-analysis by Kessels and De Haan (2003) found no significant effect of vanishing cue interventions. One reason for the lack of empirical support for vanishing cues concerns the way in which it is typically implemented: many cue fading studies involve participants generating erroneous responses early in the learning process (Riley et al., 2004; Haslam et al., 2010). As discussed previously, these produced errors require working memory resources in order to correct. This has led researchers to argue for the combination of errorless learning and vanishing cues in interventions (Riley et al., 2004; Haslam et al., 2010; Indahl, 2015), however little work has been done on the additive benefit of these two techniques in mathematics education. The present study seeks to address this literature gap by testing a novel intervention which combines the principles of error reduction and vanishing cues.

Although we have been unable to identify empirical studies which have directly attempted to employ error reduction and vanishing cue techniques to number-line estimation tasks, there is some existing work on the suitability of landmarks that might indirectly address the relative efficacy of declarative support in number-line estimation training. In the context of the number-line literature, landmarks are considered spatial or symbolic cues meant to help individuals segment the number-line into equal, meaningful pieces (Lew, 2011). Although logic would suggest that providing students with landmarks during learning should help them acquire information and reason about number-lines, there is a growing body of research suggesting that landmarks can actually be harmful to learning in some contexts. For example, a review by Lew (2011) found that there was substantial variability in the outcomes of studies when students were provided with spatial landmarks across a variety of domains. Work by Siegler and Thompson (2014) helped to shed light on why this might be the case. Siegler and Thompson (2014) provided participants with a variety of landmarks (half-way point, quartiles, deciles, etc.) during learning to help them reason about the symbolic magnitude of fractions. The results suggested that increasing the number of available landmarks can be detrimental to learners' estimation abilities when those landmarks interfere with students' ability to strategically encode helpful information about magnitude. These findings are aligned with theory proposed by Siegler and Opfer (2003), which suggests that subjective segmentation (i.e., segmentation originated or valued by the learner) is likely more helpful than overly-specific segmentation. The findings of Siegler and Thompson (2014) are also bolstered by empirical work from Ashcraft and Moore (2012) and Schneider et al. (2008) which indicate that individuals both engage in spontaneous line segmentation and are more accurate estimating quantities near their subjective segmentations. While these studies do not address the suitability of error reduction or vanishing cue techniques in the acquisition of number-line estimation skills, they showcase the existence of limitations in the efficacy of purely explicit or declarative instruction for this domain. These studies show that explicitly presenting information is not always helpful when students are learning about number-lines; this opens up the possibility that non-explicit or non-declarative techniques might be more effective at helping students improve their number-line estimation abilities.

The present study seeks to compare the relative efficacy of an intervention based on error reduction and vanishing cues and more traditional declarative instruction when it comes to helping students gain an understanding of symbolic magnitude. Specifically, we seek to answer a broad empirical question: does a number-line estimation intervention based on techniques like errorless-learning or cue-fading better help second-grade students improve their understanding of symbolic magnitude than an equivalent number-line intervention based on declarative instruction of landmarks? On the basis of prior research, we anticipate that our novel number-line intervention will result in better learning.

## Participants

Based on an a priori power estimation using an expected effect size of *d* = 0.80, α = 0.05, and 1–β = 0.80 we set a minimum sample size of 52 participants (twenty six per group). The expected effect size was based on the prior research discussed in the introduction. A total of 68 second graders from five elementary classrooms in Northeast Ohio participated in the study. Our sample size was slightly larger than the minimum sample size recommended by the a-priori power analysis due to the sizes of the recruited 2nd grade classes. The data of three participants were excluded from all analyses because either pre-test or post-test data were missing. Due to circumstances beyond the researchers' control (see description of procedures below) the study included 29 participants in the declarative group and 35 in the non-declarative group.

The classes were recruited from public schools in the university's geographic region to attend an experimental classroom in the university's center for educational technology. The participating classes typically attended the experimental classroom for one half of each school day (teacher plus class) for a period of 3-weeks. During this time, the teachers learned to use multiple classroom technologies to enhance their teaching and students were given the opportunity to explore a variety of subjects. The director of the university's experimental classroom made initial contact with participating teachers. The participating teachers then obtained written consent from the parents of participating students. All participants then provided oral assent to participate in each session of the study. The university's Institutional Review Board approved the study and all ethical standards of the American Psychological Association were followed in treatment of the participants and collection of the data.

## Design and Procedure

The original plan for the procedure was for all participants to complete a pre-test measure of symbolic magnitude on the first day (Week 1, Day 1) of their time in the experimental classroom. Following the pre-test, participants would then be randomly assigned to learn about number-lines using the non-declarative or declarative intervention. They would then complete four sessions of number line training in their assigned condition. Each session of training would take ~10 min to complete and one session would be completed each day (Week 1, Days 2–5). On the following Monday (Week 2), participants would complete a post-test measure of symbolic magnitude and on Tuesday (also Week 2) complete a measure of working memory capacity. Finally, during Week 3 participants would complete four sessions of training in the condition that they did not complete during Week 1. This was to ensure that participants were trained in both conditions and not miss the benefits of both trainings. No post-test was planned following the training in Week 3.

Due to circumstances beyond the control of the experimenters and the experimental classroom (e.g., snow days, classrooms unable to attend on a Monday or Friday, illness, etc.) the timing of the pre-test, training, and post-test s was not uniform across classrooms. Table 1 presents the training schedule for each of the five 2nd grade classrooms.

**Table 1:** Training schedules.

**Class**	**Week**	**Monday**	**Tuesday**	**Wednesday**	**Thursday**	**Friday**
Ideal	1	Pre-test	Training 1	Training 2	Training 3	Training 4
	2	Post-test	Post-test	–	–	–
1	1	–	Pre-test	Training 1	Training 2	–
	2	Training 3	Post-test	–	–	–
2	1	Pre-test	Training 1	Training 2	Training 3 and Post-test	–
	2	–	–	–	–	–
3	1	–	–	Pre-test	Training 1	–
	2	Training 2	Training 3	Training 4	Post-test	–
4	1	–	Pre-test and Training 1	Training 2	Training 3	Training 4 and Post-test
	2	–	–	–	–	–
5	1	–	–	Pre-test	Training 1	–
	2	–	Training 2	Training 3	Training 4	Post-test

The experimental classroom, in which participants completed each session, is equipped so that each participant completed all tasks on their own IBM compatible laptop computer. All measures were programed with E-Prime 2.0^®^ and presented to students on the individual IBM compatible laptop computers. Instructions were presented visually on the computers and read audibly by a research assistant at the beginning of each session. Instructions were standardized across both conditions and classrooms. At least one researcher or research assistant, two employees from the university's center for educational technology, and the relevant teacher were present during all sessions in order to maintain experimental fidelity and consistency in implementation. All researchers, research assistants, employees from the university's center for educational technology, and teachers were told to only to offer assistance to students who did not understand the presented instructions and not to assist students in completing the tasks. At the beginning of each session, one of the researchers obtained assent to participate from the students.

## Measures

In the *non-declarative learning condition*, participants are presented with 20 unique trials (per session) of a dichotomous, number-to-point estimation task meant to combine the principles of errorless learning and cue fading. For each trial in this condition, participants were presented with an Arabic numeral between 1 and 100 then asked to choose the location corresponding to that numeral from one of two provided points (red or green) on an unsegmented 0 to 100 number-line. Participants then indicated their response by pressing the corresponding key on the laptop keyboard (C for Green and M for Red). After indicating their response, participants were shown either a smiling face (indicating a correct response) or a frowning face (indicating an incorrect response). Although providing students with corrective feedback is a decidedly declarative learning technique, we elected to provide minimal corrective based on work in the discovery learning literature (another prominent non-declarative learning technique) which suggests that the absence of minimal corrective feedback can actually increase the demand placed on working memory resources (Sweller, 1988; Kirschner et al., 2006) and lead to harmful misconceptions (Klahr and Nigam, 2004; Kirschner et al., 2006). Given that our stated goal is to employ non-declarative learning techniques as a means of reducing working memory demands for young students, we elected to provide minimal corrective feedback.

**Figure 1:**
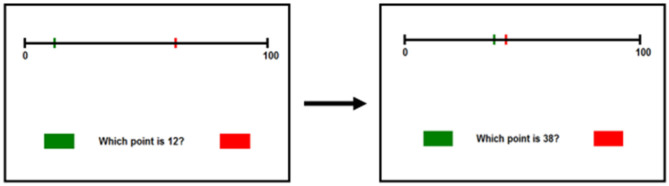
Stimuli for the non-declarative learning condition. The task begins with the red and green lines separated by extensive spatial distance. As the task progresses, the lines become closer together and more difficult to discriminate.

To reduce errors made during initial learning, the red and green points (representing response options) were spatially distant from one another during early trials (e.g., target is 15 and points represent 15 and 75); this large spatial distance was meant to facilitate easy discrimination between response options and it was expected that students would make minimal errors on these trials. This assumption was supported by pilot data, which indicated that fewer than one in five students made any errors during the errorless trials. As the intervention progressed, the red and green response options become closer together in space (e.g., target is 15 and points represent 10 and 15); this was meant to instantiate the vanishing cue technique, as the cue of spatial distance loses its discriminatory power and becomes less helpful as the task progresses. As such, participants must generate their own subjective landmarks or strategies to correctly complete the task. Each training session took ~10 min and used unique Arabic numerals which were randomly drawn from across the 0–100 range. No values were presented more than once either within or across training sessions. We excluded numerals ending in 0 or 5 to avoid having response options fall directly on provided landmarks in the declarative condition. Examples of the non-declarative learning stimuli can be found in Figure 1.

**Figure 2:**
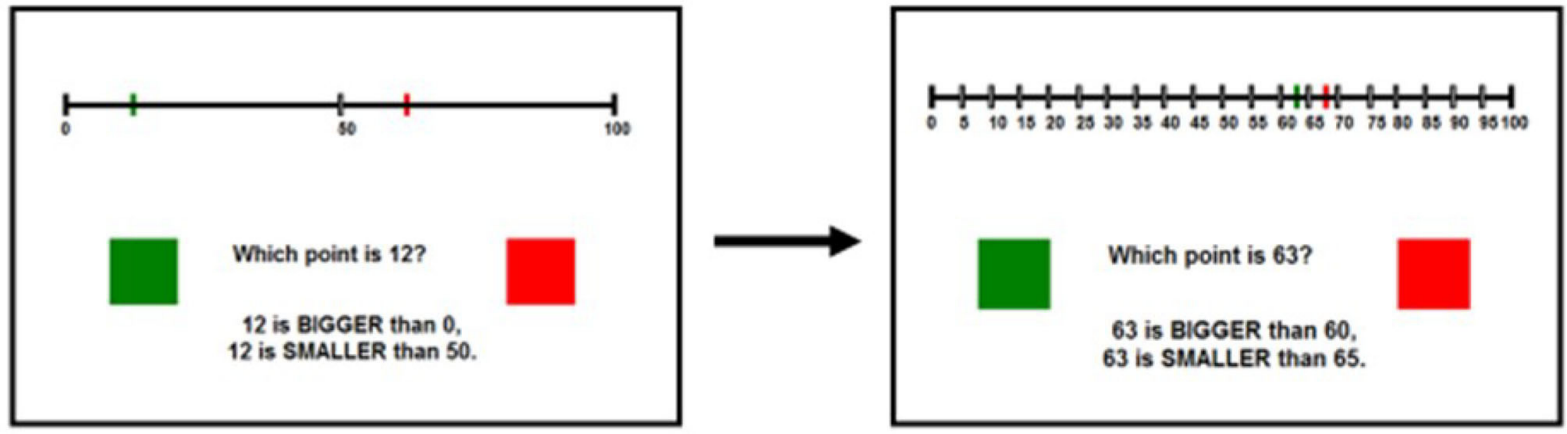
Stimuli for the declarative learning condition. The task follows the same spatial fading as the non-declarative condition but this fading is offset by an increase in available declarative cues.

In the *declarative learning condition*, participants were also presented with 20 trials (per session) of a dichotomous, number-to-point estimation task. As in the non-declarative learning condition, participants in this condition were presented with an Arabic numeral and instructed to choose the correct location from two provided points (red or green) on a 0 to 100 number-line. Responses were indicated in the same manner as in the non-declarative condition and the same minimal corrective feedback was provided. Just like the non-declarative learning condition, the initial trials of each session featured red and green response points which were far apart in space and then got closer together as the session progressed. Unlike those in the non-declarative learning condition, participants in the declarative learning condition were given two forms of declarative support to help them cope with the incremental loss of the spatial cue. First, in addition to the labeled end values of 0 and 100, participants in the declarative learning condition were also provided with landmarks on the number-line to support identification of the correct response (e.g., labeled marks at halfway point, quartiles or deciles). These landmarks were adaptive to task demands; as the red and green points became closer together, the provided landmarks increased in specificity. Second, participants were also provided with declarative support (in the form of onscreen text) about the presented numeral's relationship to two of the presented landmarks on the number line (e.g., 15 is bigger than 10 but smaller than 20 with decile landmarks presented). Thus, as the proximity between the red and green points increases throughout the task, participants will receive increasingly specific landmarks and specific declarative support regarding the use of those landmarks. The first five trials in this condition provided landmarks and declarative support based on the midpoint, the next five trials used quartiles, the next five trials used deciles, and the final five trials used landmarks in increments of five. Each training session took ~10 min and used unique Arabic numerals which were randomly drawn from across the 0–100 range. No values were presented more than once either within or across training sessions. We excluded numerals ending in 0 or 5 to avoid having response options fall directly on provided landmarks in the declarative condition. Items in both training conditions were presented in the same pseudorandomized order, distance, and duration. Examples of the declarative learning stimuli can be found in Figure 2.

The outcome measure for this study was a *number-line estimation task*. Specifically, participants completed a point-to-number number line estimation task, which is thought to indicate knowledge of symbolic magnitude. For each trial of the number-line estimation task, participants were presented with an unsegmented 0 to 100 number-line with a single black mark somewhere on the number-line. Participants were asked to type the Arabic numeral that they believed the mark represented. This task was administered as both a pre-test and a post-test, although a different set of stimuli were used for each test. The pre-test and post-test each consisted of 40 unique trials and lasted ~15 min. No corrective feedback was provided during this task. While the pre-test and post-test used different numbers from each other (i.e., 40 unique correct numeral responses for the pre-test and 4 unique correct numeral responses for the post-test), all values used in the outcome measure were presented once during training. Stimuli were selected for the outcome measures to ensure an even sampling across all four training sessions in an attempt to reduce a possible recency bias in the post-test.

**Figure 3:**
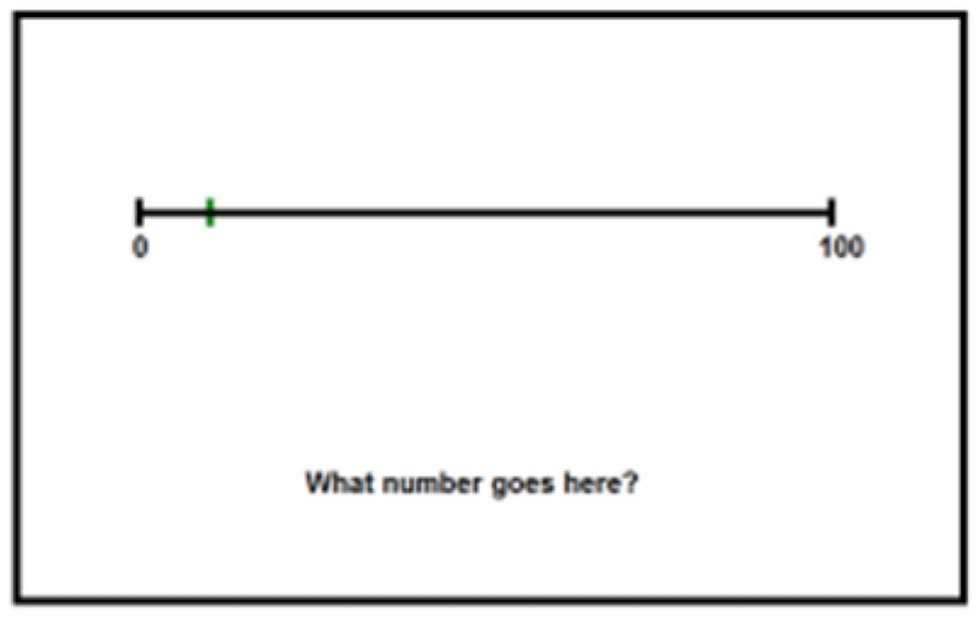
Stimuli for the pre-test and post-test tasks. Participants are given a single point on a number-line and must type the symbolic number which matches the position of the point.

This outcome measure was specifically chosen to be methodologically distinct from both training tasks in order improvement based solely on practice effects. The training tasks each use variant of the bounded, number-to-position estimation task. This is the most common instantiation of the number line estimation task in the published literature and requires participants to select a location on a number-line which corresponds to a presented numeral. The outcome measure is a bounded position-to-number task where participants are presented with a location on a number-line and must produce the corresponding numeral. These tasks variants require different responses from participants (selecting a spatial location vs. producing a symbolic numeral) and vary in the number of possible responses (two possible locations during training vs. 100 possible symbolic numbers on the outcome). The current consensus of the number line literature seems to be that these task variations likely measure the same underlying construct (symbolic magnitude representations) but should not be treated as isomorphic or interchangeable due to potential variations in difficulty or strategic behavior (Siegler and Opfer, 2003; Schneider et al., 2008). Examples of the number-line estimation task stimuli can be found in Figure 3.

## Results

**Figure 4:**
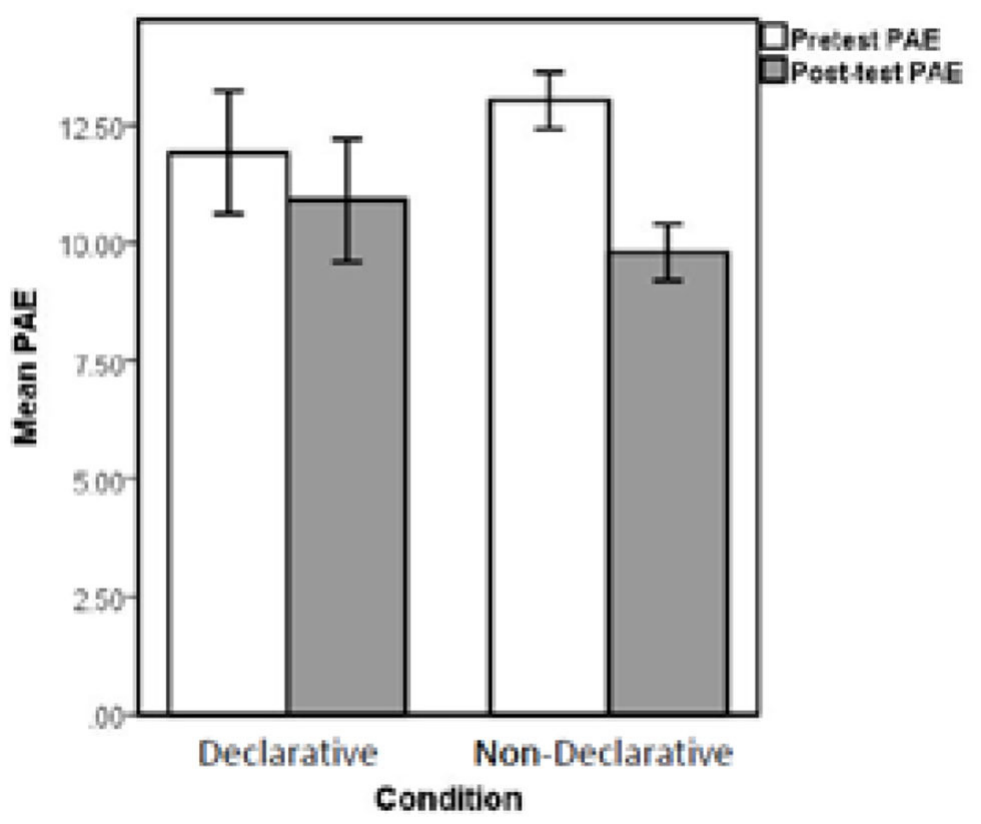
Mean PAE for pre-test and post-test performance by experimental condition.

The dependent measure of interest was the percent absolute error (PAE) on the post-test. PAE is calculated as (|correct response—participant response|)/100. Table 2 presents the means, standard deviations and *t*-test results of pre-test and post-test PAE by class and treatment condition for all participants. Figure 4 presents group PAE means by test. Descriptive statistics revealed the presence of one prominent outlier in the declarative learning condition. As a result, analyses were computed both with the full data set and with the outlier removed. Removal of the outlying individual did not change the pattern nor significance of the reported effects. As such, all analyses reported below were calculated with the full data set.

**Table 2:** Effect size estimates for CCMA.

	**Declarative**			**Non-declarative**			**Comparison**			
**Class**	**Mean PAE**	**SD**	***n***	**Mean PAE**	**SD**	***n***	**s_**pooled**_**	**T**	**SE**	***p***
1	17.86	8.90	6	9.30	4.24	9	6.45	2.52	3.40	0.03
2	7.53	2.53	10	7.51	5.34	6	3.78	0.01	1.95	0.99
3	4.99	0.49	2	9.18	5.56	5	4.98	−1.1	4.16	0.36
4	12.58	7.15	6	9.48	4.31	9	5.58	1.06	2.94	0.31
5	19.15	12.88	5	8.83	3.88	7	8.68	2.03	5.08	0.07

Due to the uneven training schedule, we conducted a continuously cumulating meta-analysis (CCMA; Braver et al., 2014) on the data treating each class as its own separate study. The CCMA is typically used to determine the pooled effect size of a given intervention or grouping variable across multiple studies; it can be thought of as a way to assess the strength of an effect across variations in methodology, materials, or sample characteristics. Although the CCMA is typically used to determine the pooled effect size from different studies, we used it to assess the pooled effect of our experimental intervention across the variations in treatment administration, testing, and class characteristics. In this way, each of the classrooms that participated in our experiment was treated as its own unique study. To compute the CCMA, we first conducted an independent-samples *t*-test to compare group means on both pre-test PAE and post-test PAE for each classroom (see Table 2 for results). We then pooled the effect size estimates produced by each of these analyses into a single meta-analytic index for pre-test and another for post-test. The CCMA results for pre-test PAE (based on the pooled Cohen's d and fixed effects) indicate that there were no group differences at pre-test, pooled *d* = 0.18, *SE*_*pooled*_ = 0.25, *p* = 0.47, 95% CI [−0.31, 0.68]. This suggests that the groups could be considered functionally equivalent, in terms of symbolic magnitude estimation skill, before the intervention. The CCMA results for post-test PAE (based on the pooled Cohen's d and fixed effects) indicate that there were significant differences between the groups at post-test. Specifically, the declarative learning group had larger PAE's (M = 14.25, SD = 15.15) than the non-declarative learning group (M = 9.39, SD = 4.59) producing a pooled *d* = 0.52, *SE*_−*pooled*_–= 0.26, *p* = 0.046, 95% CI [0.01, 1.03]. Pooled Cohen's d can be interpreted as the standardized mean difference between groups computed across multiple studies. Our pooled Cohen's d of *d* = 0.52 can be considered a medium effect size (Cohen, 1988).

As the CCMA indicated a significant effect at post-test despite variations in training implementation, we aggregated pre-test and post-test data then conducted a 2 (pre-test vs. post-test) within subjects × 2 (group: declarative vs. non-declarative) between subjects ANOVA on mean PAE. The results indicate that the main effect of time (pre-test to post-test) was not significant, *F* < 1, yet there was a main effect of group, *F*_(1, 64)_ = 4.80, *p* = 0.032. The key finding was a significant interaction, *F*_(1, 64)_ = 5.10, *p* = 0.027. These results suggest that, although the two groups were nearly identical in PAE at pre-test, students in the non-declarative learning group performed significantly better at post-test than those in the declarative learning group.

Lastly, in order to better understand the relationship between training condition and position of the presented stimuli on the number line we divided the line into four equal segments and conducted a linear regression. The regression analysis was designed to predict post-test PAE using group and number-line segment as predictors and including an interaction term between segment and condition. The results of the regression analysis [*R*^2^ = 0.07, *F*_(3, 95)_ = 2.68 *p* = 0.05] indicate that there was no significant effect of segment (β = *0*.42, *t* = 1.40, *p* = 0.16), nor of the interaction (β = 0.36, *t* = 1.19, *p* = 0.24), but there was a significant effect of condition (β = 0.24, *t* = −2.44, *p* = 0.02), indicating the non-declarative learning group had smaller percent absolute errors than the declarative learning group for each segment of the 0–100 number-line.

## Discussion

In accordance with our hypotheses, the results of the present study indicate that students who learned about number-lines using an intervention based on the principles of errorless learning and vanishing cues had better representations of symbolic magnitude than those who learned about number-lines with substantial declarative support. Importantly, we found these effects despite differences in intervention implementation and fidelity. Although it must be stressed that these data represent only a modest first step toward answering the questions set forth in the introduction and additional work is needed to replicate these findings, the present study provides preliminary evidence in support of using a combination of error reduction or errorless learning and cue-fading techniques in early STEM education. This is in line with the conclusions of Indahl (2015) which found that interventions which use errorless learning and vanishing cues can help eighth-grade students learn to factor polynomials. The results of that work found that students who learned to factor polynomials were more efficient at factoring novel polynomials than those who learned about polynomial factoring from a more traditional lecture. However, these results did not replicate for tests of declarative rule knowledge; many of the students who learned how to factor polynomials from the non-declarative intervention were able to factor polynomials without being able to articulate the rules or mathematical procedures for factoring. In conjunction with our results, these findings open up interesting future questions regarding the qualities of knowledge representations produced by the combination of errorless learning and cue-fading techniques.

## Limitations and Future Directions

The lack of methodological consistency and fidelity makes more fine-tuned analyses difficult at the present time. Future studies should attempt to replicate and extend these findings using a larger sample size and more methodological consistency. These materials should also be evaluated in student populations with more variability in background knowledge, math anxiety, working memory, and developmental differences. Additionally, the present study used a relatively immediate post-test with limited possibilities for transfer. Future studies should examine both the long-term effects of non-declarative training (compared with declarative instruction) and the extent to which non-declarative training impacts transfer to a wide range of numerical skills and math achievement. Finally, additional research is needed to determine which areas of mathematics are amenable to non-declarative learning techniques and which topics require direct instruction. Nonetheless, this study represents compelling early evidence for the suitability of errorless learning and cue-fading techniques in educational settings.

## Data Availability Statement

The raw data supporting the conclusions of this article will be made available by the authors, without undue reservation.

## Ethics Statement

The studies involving human participants were reviewed and approved by Kent State University IRB. Written informed consent to participate in this study was provided by the participants' legal guardian/next of kin.

## Author Contributions

EG was responsible for designing and programming the intervention, collecting data, and most writing. CW was responsible for supervising data collection, data analysis, and editing.

## Conflict of Interest

The authors declare that the research was conducted in the absence of any commercial or financial relationships that could be construed as a potential conflict of interest.
